# Hospital and Institutional News

**Published:** 1916-02-19

**Authors:** 


### HOSPITAL AND INSTITUTIONAL NEWS.

### LONG LIFE IN PERSONAL SERVICE.

It is a notable coincidence that the late Clara Barton, known in America as "The Angel of the Battlefield," whose most interesting life Macmillans have just published, should have lived until her ninetieth birthday, as did Florence Nightingale also. Both carried their lives in their hands during the arduous period of the wars in which they lent their invaluable aid to the sick. Both came triumphantly through the many and great dangers they voluntarily faced. Afterwards both lived every hour of every day to the end of their lives. The review of Clara Barton's life reached us in the week the Florence Nightingale Memorial was unveiled by Her Majesty the Queen.

### THE MEDICAL PROFESSION AND THE WAR.

The outstanding event of the past week as regards the demands made by the war upon the medical profession is the revolt which has been in progress against the Central Medical War Committee, but is now subsiding as the result of interviews with Mr. Tennant and with the Central Committee. Several letters have appeared in the daily Press for and against the schemes propounded by that body; and as some of the statements made have been of doubtful validity, it may be of service tc re-state briefly the position of the Committee and the basis of some of the dissent which its proceedings seem to be arousing. The Committee, which had originally the word "Emergency" as part of its name, originated with the British Medical Association, whose Council, if we remember rightly, nominated several members to help in (medical) recruiting. Other members from outside the British Medical Association were co-opted on to the Committee, whose joint secretaries were the secretary of the British Medical Association and an ophthalmic consulting surgeon. It will thus be seen that the Committee was not in any sense elected by the medical profession: it was to all intents and purposes self-elected. The medical profession, however, is hardly entitled to throw this in the teeth of the Committee: something had to be done for filling up the requirements of the R.A.M.C., and no one (in any collective or organised fashion) stirred a finger to do it except the Central Medical War Committee.

### THE CENTRAL MEDICAL WAR COMMITTEE.

This Committee has for months past been distinctly active. It was recognised by Government for the purposes of Lord Derby's scheme; and it has for a long time negotiated direct with the Director-General of the Army Medical Service, Sir Alfred Keogh. It has done distinctly good service in drawing the attention of the War Office authorities to those numerous cases of waste or misuse of medical officers, such as recently formed the subject of many letters to the *Daily Telegraph*; and there is reason to believe that several overdue reforms have been initiated in consequence of its representations. In pursuance of its officially recognised endeavours to fill up the commissioned ranks of the R.A.M.C., the Committee has invited every medical man under forty-five to register his name as willing to take a commission when called upon to do so by the Committee, which undertakes to adjudge from his circumstances whether or not he is, or is not, more required in the Army than in civil life. Further, the Committee has published a classification of the medical profession, by which each individual can see how he stands: thus single men precede the married, men with partners not at the war go before men in single-handed practice, and so forth. If every medical man agreed to this scheme of registration, many and great advantages would certainly be gained; but there are several reasons why this unanimity has not been attained, and is not likely to be.

### EXPRESSIONS OF DISSATISFACTION.

To begin with, many medical men feel that the Committee is too pliant in the hands of the Director-General. They hold that Sir Alfred Keogh's estimates of the needs of the Army should not be accepted without inquiry: that from the nature of things he cannot assess either the terrible financial difficulties of men in practice called upon to leave all and join the Army or the serious hardships which will overtake the civil population when deprived of the most active sections of the medical profession. They feel that the Committee has no efficient means of ensuring that due economy will be used and that no more doctors will be called for than is absolutely necessary. Then, they feel doubts about the Central Medical War Committee itself: a number of justly respected senior members of the profession are on that Committee, but there are rumours that they do not attend its meetings and are not even furnished with notices and agenda of such meetings. Rumour is, we know, a lying jade; and we should not mention such unsubstantiated statements were it not that they are in many men's minds and have distinctly lessened the confidence of the profession in the Committee. If they are, as may be hoped, quite baseless, the sooner this is made convincingly clear to the medical profession the better for all concerned. Possibly they arose from the fact that some of the more active spirits in the Committee are men of admitted energy and eloquence, but of less repute for sagacity and statesmanship.

### MEETINGS AND DEPUTATIONS.

At all events the Committee has been established so long that opposition to it now is a thankless task. Nevertheless, opposition there is, and it has come to a head in a series of meetings during the past week. The principal contentions of this opposition are that the whole profession, instead of those under forty-five alone, should be organised and utilised; and that this should be done under some system of compulsion, not by a voluntary agency which cannot compel the obedience of recalcitrants. It is pointed out that if the young unmarried men and others in the early "groups" of the Central Committee's scheme refuse to register, there is no way of making them do so; and the upshot will be that the middle-aged married man in single-handed practice may find himself taken for service before the unmarried, the junior officers of institutions, and others rightly placed in the early "groups." That this may be the case is obvious on the face of matters; and it explains much of the dissatisfaction which has become vocal during the last few days. The Director-General's position is said to be, "If you don't like this Committee, elect another one: if you don't make your own arrangements to supply the men I want, compulsion will be the result, and you may not like it." The best solution of the difficulty, in our opinion, would be the recognition by the Central Medical War Committee that their schemes are open to legitimate criticism, and the election of three or four of the other side to their deliberations.

### SECURITY OF TENURE FOR MEDICAL OFFICERS OF HEALTH.

A reform which has been demanded for many years seems at length to be within sight. At present most medical officers of health are appointed for a stated period, and their appointments are subject to renewal by the sanitary authorities at the end of such periods. In London the medical officers of health can be dismissed only with the consent of the Local Government Board, and similar security of tenure was given to county medical officers of health by the Housing and Town Planning Bill of 1909. These special classes form, however, a comparatively small proportion of the total number of medical officers of health. As long ago as 1871 the Royal Sanitary Commission recommended that in order that these officers might be able to discharge their duties without fear of personal loss they should not be removable by any local authority except with the sanction of the central authority. This recommendation has been repeatedly endorsed by other Commissions and Government Committees. The Land Inquiry Committee in 1913 expressed the opinion that the Housing Acts will not be properly enforced until the medical officers of health obtain a greater security of tenure. The Local Government Board has now prepared an Order embodying this reform, which applies also to inspectors of nuisances. Mr. Long is, however, of opinion that, owing to the dislocation due to the war, it will not be advisable to issue the Order at present. Medical officers of health and the public are to be congratulated upon this successful end to a long-fought dispute.

### SECRETARYSHIP OF THE NORFOLK AND NORWICH HOSPITAL.

Mr. Frank Inch, secretary of the Walsall and District Hospital, has been appointed to the secretaryship of the Norfolk and Norwich Hospital from amongst 206 applicants for the position. Nine candidates were interviewed by a sub-committee of the board on the 4th inst., and four of these were selected to appear before the board on the following day, including Mr. Inch, a barrister, a schoolmaster, and one other candidate. Mr. Inch's career has been devoted entirely to work in hospitals. He commenced his work at the Bristol Royal Hospital for Sick Children, where he occupied the position of clerk for eight years. From this institution he obtained a clerkship at King Edward VII.'s Hospital, Cardiff, and two years later was appointed assistant secretary there, which position he filled for two and a half years, until his appointment to the secretaryship of the Walsall and District Hospital, where during the past three years he has rendered excellent service. Mr. Inch is an enthusiastic hospital worker; his training and fifteen years' association with various types of hospital management should fit him for the important position he has obtained at the Norfolk and Norwich Hospital, Norwich.

### A STRIKE AT BRADFORD.

At the Bradford City Council meeting on Thursday, February 10, reference was made to a strike of the entire medical and nursing staff, numbering twenty-three, at the Babies' Welcome, as a protest against the action of the Council in refusing to advance the salary of Dr. Helen Y. Campbell, medical officer of the department, from £350 to £450 a year. It was stated that Dr. Campbell, whose ability and excellent work had been the subject of eulogy by the President of the Local Government Board, was paid less than any doctor in the Corporation service, and had worked for a year on the strength of a promise that her salary should be increased as stated. In the meantime she had refused many better offers—some at greatly enhanced salaries. The Council still refused to sanction an advance, and failing the success of efforts to induce the staff to withdraw their resignations the department may have to be closed. The opponents of the increase of Dr. Campbell's salary urged that it should be held over until after the war. Bradford is not going the right way to attract capable officials to its service; it managed to raise over £30,000 in three weeks for a war hospital, and seems to be spoiling its ship for a ha'p'orth of tar.

### THE ROYAL INFIRMARY, EDINBURGH.

The annual report by the managers of the Edinburgh Royal Infirmary discloses an excellent account of work done and progress maintained. The most important passages are those dealing with the ordinary income of the charity, which was nearly 30 per cent. more than in the previous year: the actual increase reached the large sum of £10,141. This very gratifying and substantial rise in ordinary income was due to a special appeal issued by the managers, and it is to be hoped that this year will show no falling off, though it is stated that many of those who gave, or who increased their usual gifts, did so expressly for the appeal year only. As showing what energy and initiative can do, it may be mentioned that one lady, Miss Trotter, of Colinton House, raised £3,685 for the institution by organising and carrying out a Badge Day. The Edinburgh Infirmary managers have evidently their own opinions about a point which has been a good deal discussed by institutional secretaries and governors—namely, the writing down of depreciated investments. This question has been ventilated in The Hospital before now, but unanimity has not yet been secured. At Edinburgh investments are evidently never written down: thus different holdings of Consols are shown at all sorts of prices between 80 and 98, clearly the prices ruling when the respective blocks of stock became the property of the institution. There is, however, in our own view much to be said for the writing-down policy, at any rate in peace-time.

### THE NURSING STAFF AND A TEST OF EFFICIENCY.

It is instructive to remind the public how closely the conditions of nursing affect administration in our special hospitals. At St. Mark's Hospital for Cancer, Fistula, and other Diseases of the Rectum, for instance, the latest report shows that the most pressing difficulty is in regard to cases of cancer, which always throw a heavy burden on the nursing staff, and also require expensive treatment; so that if the funds are maintained more cases of rectal cancer can be treated, and prompt treatment is perhaps the chief help which such cases demand. Another aspect of the question is the strain imposed on the nurses in charge of these cases, and Sir Richard Biddulph Martin, the honorary treasurer, emphasised the fact that they paid the greatest possible attention to the comfort of the nursing staff. Particularly in such a hospital as this, efficiency of the nurses very largely depends upon the consideration and the comfort which they enjoy. On this ground alone, therefore, an intelligent donor will always satisfy himself of the attention paid to the nurses, when estimating the claims of such a hospital to his support.

### A RAPID REORGANISATION.

The difficulties which preceded the decision to extend the war hospital at Bradford have been surmounted with great promptitude. The large sum of money required—namely, £30,500—was practically raised within three weeks, and while the Lord Mayor, Mr. Thomas Howarth, was throwing his energies into this side of the question, the Guardians were hardly less prompt in preparing the plans for the new buildings. The extension of St. Luke's Hospital has been achieved so far as a result of mutual accommodation by those responsible for the new scheme, and it did not prove necessary even to issue a regular public appeal. If this is not making up for lost time, it would not be easy to find an instance of it.

### AN HONORARIUM FOR THE HONORARY STAFF.

At the Norfolk and Norwich Eye Infirmary, according to the latest report, an arrangement has been made with the honorary medical staff which may be worth recording. A sentence in the report, which was read at the recent annual meeting by the woman secretary, Miss T. Burton, describes the arrangement thus: "During the year 579 soldiers have received treatment at the infirmary, thus placing a great amount of extra work upon the honorary medical staff; and, in view of this, the committee have voted the staff an honorarium—a step which, the committee believe, will meet with the approval of the governors." It did; for the report was at once adopted. It would be of interest, however, to other institutions to have the details of the arrangement made clear, since, though salaries have been frequently raised in the case of residents, it has not been usual to vote an honorarium to the "honoraries." Another noteworthy change at the institution has been the employment of a professional accountant to audit the books. The adoption of this plan is stated to have been due to a suggestion of the honorary auditors; one speaker put it that for the first time in the history of the charity, so far as he knew, the accounts had been audited by professional accountants. Of the advantage of this departure there can be no question; but as the hospital was founded in 1822 it has been some time in coming about.

### THE CUT AND COLOUR OF HOSPITAL CLOTHES.

The hospital suits issued to patients in the Voluntary Aid Detachment hospitals have been severely criticised, particularly at Cambridge, where the County Director, the vice-presidents, and commandants of this branch of the British Red Cross Society have applied to the Surgeon-General for an issue of the ordinary blue hospital suits. This is not the first time such an application has been made, but it has generally been refused on the ground of an insufficient supply of the material. Grey clothing has been permitted, but this is what is objected to so strongly; on the ground, it was urged, that the cut of the clothes made them resemble pyjamas! The colour, though apparently not very popular, was not so much criticised as the cut, or absence of cut. So keenly is this defect felt that one speaker at a meeting of Cambridge commandants suggested starting a fund to provide the men with an alternative to "this terrible dress." Can any valid reason be suggested why men in the V.A.D. hospitals should not be provided with the customary blue hospital suit? We pause for a reply; and invite our friends the editors of the new Red Cross hospital *Gazettes* to supply it.

### MORE INFIRMARY BEDS FOR SOLDIERS.

The Metropolitan Asylums Board, acting on a request from the Local Government Board, is opening two additional institutions for sick children—namely, Cleveland Street Infirmary, Westminster, and Park School, Hanwell. Sir Arthur Downes, Medical Inspector of the Local Government Board, in informing the Metropolitan Boards of Guardians of these arrangements, points out that their object is to relieve the pressure on the infirmaries, so that the accommodation for wounded soldiers may be increased. Looking beyond the exigencies of the moment, the matter is important as indicating the further departure of the M.A.B. from the original restrictions on its usefulness.

### "SHELL-SHOCK" AND SUICIDE.

Notwithstanding the painful nervous symptoms of the medley of nervous diseases which are collectively, but most inaccurately, spoken of as "shell-shock," very few cases of suicide have been reported amongst the sufferers; and, in fact, cases of suicidal tendencies are distinctly rare amongst the sufferers from these neurasthenic and mental disorders. As an exception to this rule may be noted the recent suicide of Lieutenant-Colonel J. W. Stokes, an officer of the (Territorial) Royal Army Medical Corps, who since his departure for France last April had been twice sent home for "shell-shock." It is not stated in the report of the inquest with what unit he had been serving; but the rank of the deceased officer alone is sufficient guarantee that he was not serving in the trenches, and in all probability he would be seldom, if ever, directly under fire. Most likely an unstable nervous system was over-strained by the sights and sounds of modern warfare; and the verdict of suicide during temporary insanity probably expresses the facts of this distressing case much more nearly than the diagnosis under which he had been invalided from France.

### THE DRUG HABIT AMONG SOLDIERS.

Two cases were before the Court last week which furnish testimony that the habit of drug-taking exists in the Army to no inconsiderable extent. In one case there was evidence to show that at Folkestone certain persons were carrying on an illegal traffic in cocaine, supplying this narcotic substance to soldiers of a Canadian regiment; while in the other it was shown that there is a considerable demand among members of His Majesty's Forces for morphine prepared in a suitable form for administration at any time. Both these cases reveal a condition of affairs which the Army authorities will no doubt take immediate steps to remedy. The clandestine traffic in cocaine, however, is no easy matter to deal with, and short of making it a criminal offence for any manufacturing chemist or dealer to supply cocaine to any person, except on the written order of a duly registered medical practitioner, it is difficult to see how the evil can be stopped. So long as so many sources of supply are available to scoundrels with no conscience and bereft of all sense of patriotism, so long shall we find people willing to take the risk of making large profits out of the folly of others.

### THE SALE OF MORPHINE TO ARMY OFFICERS.

It will fortunately be less difficult to cope with the danger arising out of the sale of morphine in the manner disclosed in the course of the hearing of proceedings in the London police courts against one of the largest of the departmental stores, and a firm of London chemists of high repute. From the facts elicited in the course of the hearing of these cases it appears that small gelatine squares, or "lamels," containing morphine are reaching the troops on active service. These "lamels" are prepared for sale in little packets of twenty-four, the whole containing four grains of morphine hydrochloride, the packets being of a convenient size to place in a soldier's pocket-book. In the case against the stores, counsel for the defence admitted that there was a great demand among officers for these "lamels," but, as was pointed out by the prosecuting counsel, they are dangerous things to be in the hands of men on active service who know nothing about drugs, and it is conceivable that through taking one of these "lamels" a soldier might sleep on duty. The practice was brought to light through the discovery of a technical breach of the Pharmacy Acts; but it seems to us that the sale of these articles is undesirable even when all the formalities of the Pharmacy Acts are observed.

### WANTON PROCEDURE OF ALEXANDRA DAY MANAGERS.

We agree with the remark, lately made by Sir C. Ernest Tritton at a Council meeting of the Metropolitan Hospital Sunday Fund, that it is unfortunate that Alexandra Day has put itself in collision as to dates with the Sunday Fund this year. The third Wednesday in June has been most improperly chosen by the Alexandra Day Committee, for it falls in the week throughout which the collection for Hospital Sunday is to be made. The hon. secretary of Alexandra Day states that this third Wednesday in June has "always been" their day, but since Alexandra Day is a mushroom growth and the Sunday Fund the father of all charity days whatever, it is clear who has the real priority of place if there be any risk of clashing. No such clashing has arisen in the past, and it is condemnatory of the managers of Alexandra Day that they should have created this collision, which may prove to their day disastrous if the public appreciate the fact.

### A POOR-LAW MEDICAL OFFICER'S PROTEST.

Dr. Lock, the medical officer to the Uxbridge Union Workhouse, has written to the Local Government Board protesting against being required to look after sixty inmates in the Uxbridge Workhouse, boarded there by the Richmond Guardians. The Richmond Guardians have contracted with the War Office to receive sick and wounded soldiers in their infirmary. In order to make room for them they have arranged to send a number of their indoor poor, not exceeding one hundred, to the Uxbridge institution. Dr. Lock is a part-time medical officer, and he applied without success to the Uxbridge Guardians for extra remuneration for the work thus thrown upon him. The point he raises is a very important one. As he rightly points out, he was appointed to give medical attendance to patients chargeable to the Uxbridge Union. The Richmond Guardians are responsible for the care of their poor, and they should either appoint a medical officer of their own to look after them, or should pay a capitation fee to cover the cost of medical attendance. Either the money paid by the Richmond Guardians to the Uxbridge Guardians covers only the cost of their maintenance, and the Richmond Guardians are escaping from their liabilities, or the amount paid does include the cost of medical attendance, in which case the Uxbridge Guardians are making a profit at the expense of their medical officer. The decision of the Local Government Board will be awaited with interest by many Poor-Law medical officers who are in a similar position to Dr. Lock.

### THE "RELIGIOUS RIGHTS" OF THE WOUNDED.

Till lately it seemed as if the new hospitals for the wounded would not have to count among their troubles what is generally described as the religious difficulty. But the illusion of complete immunity has been dispelled. At the Red Cross Hospital at Knutsford difficulties have arisen concerning the right of the men to attend services outside the institution. The matter was raised by a protest from the local Roman Catholic priest, who expressed himself anxious to secure the "religious right of the most vital kind." The Lady Commandant, whose courtesy in informing him of the arrival of Roman Catholics the priest acknowledged, wished to have a clear direction from the authorities, and the matter has been settled by granting permission to attend services outside to any men who wish to do so, on condition that they give notice of their wishes to the Commandant a day in advance. The difficulty, therefore, has been settled; but while no sane person wishes to add to the rules and regulations which already surround the soldier, we have some sympathy for the hospital authorities, themselves to a considerable extent under discipline, in refusing to sanction the practice until official permission had been obtained.

### ILLUSTRATORS AND WRITERS ON THE NEW HOSPITAL "GAZETTES."

The February number of the *Gazette* of the 3rd London General Hospital, Wandsworth, is again chiefly remarkable for its illustrations, the best of which is Pte. C. R. W. Nevinson's "The Receiving Hall: A Futurist's Impression." Among the numerous humorous sketches, Pte. G. F. G. Fisher's two illustrations of the patient's point of view deserve mention, as also do some observant portraits by Pte. C. F. Evans, which have a bite and humour of their own. While it is noteworthy that "all the contributions to this number, without any exception, are said to "come from within the hospital," the letterpress is less original than the drawings. Only a writer who is an expert in humour can say exactly what he wants to say without being dull or causing misunderstanding; but the draughtsman's pen has this advantage over the average writer's, in that lines do not betray their secrets to the public as words, except in practised hands, may do; and the writer is more restrained in consequence. The distinction is worth making, since the life of these hospital *Gazettes* must be the spirit of comedy; and as that spirit is either keenly critical, or nothing, lively writers may be silenced by lack-humour disguised as Authority. This is why many a medical students' *Gazette* is a censored organ; and, therefore, worthless. But illustrations disguise better the criticism which alone can give them life. It is odd, when one thinks of it, but many a hospital *Gazette* would welcome Hogarth or Tenniel, or even Dyson or Raemaekers, which would turn Swift or Molière or the author of "Hudibras" out! Posterity laughs with these great writers, but contemporary Authority is rarely on their side.

### THE POOR-LAW MATTRESS.

Now that the Local Government Board by publishing reports on the undesirable character of flock for mattresses, pillows, and bolsters has led to the condemnation of this material in all institutions whose standard of administration is up to date, the question of substitutes is receiving deserved attention by local authorities. As one instance out of many, we may note the recent decision of the Reigate Board of Guardians in making provision for mattresses and bedsteads in their new infirmary. After considering a variety of tenders the house committee has recommended the purchase of thirty-two mattresses, made of hair and wool, at a cost of 31s. 9d. each. The committee have also been considering samples of "Lyxhayr," a material which our readers have had placed before them in our columns. The committee's statements in regard to it are worth quoting. "Inquiries," they state, "had been made of the Epsom, Reading, and Farnham Unions, where these mattresses were used, the last-named stating that with a few exceptions these mattresses were used both in the house and infirmary. The cost of a 4½-inch mattress was 23s. 9d." Therefore, "as an experiment," the committee recommended that twelve of these mattresses be purchased of Messrs. Dollery and Palmer, London. In view of the cost of the hair mattress on the one hand, and of the desirability of replacing flock by a substance which possesses many of the virtues of hair, the successful experience with a substitute like "Lyxhayr" deserves attention by all institutional managers.

### THE OVERWORKED HEALTH VISITOR.

The Borough of Dudley is reconsidering its arrangements for health visiting, as a result of the suggestions made by Dr. Hutchinson, of the Local Government Board, to the Town Clerk. He pointed out that there was too much work for the present health visitor to undertake single-handed, and that two health visitors were required if all that there was to do were to be properly done. Dr. Hutchinson has put forward two alternatives. The one is to double the present health-visiting staff, and to divide the town into two areas, with a health visitor placed in charge of each. The other is to divide the town into three areas, and to amalgamate health visiting and school nursing, in which case another health visitor would have to be appointed. As the Local Government Board would make things easy for the Corporation by paying one-half of the cost of the two nurses, there should be no difficulty in the way of one of these plans being carried out. Health visitors, as a new class of officer which is only now receiving adequate recognition, are overworked and underpaid. To cope with the work a proper staff is necessary, and now that the work itself is being entrusted to trained persons, which was not always the case, the time has come to insist on salaries at the full rates for professional work.

### THE ROYAL SANITARY INSTITUTE.

We have received a syllabus of the lectures and demonstrations arranged by this well-known teaching institute, which reaches this year its fortieth anniversary. Among those courses likely to be of interest and value to institutional workers we note that commencing on February 21, designed especially for students of school hygiene, women health visitors, tuberculosis visitors, and school nurses. It consists of lectures and practical demonstrations on physiology, personal hygiene, the sanitation of school buildings and dwellings, the hygiene of child-life and educational methods, and tuberculosis. The lectures are at 6 p.m. on Mondays and Fridays up to April 10, and are delivered by six different lecturers, who are experts in the topics allotted to them. An interesting and novel feature of the course is the arrangement whereby each student will attend six infant consultations conducted by a well-known specialist in this branch of work. There are also demonstrations in the museum of sanitary appliances owned by the Institute, visits to public works and other places of sanitary interest, and the use of a reference library, lending library, and reading-room for an inclusive fee of one guinea for the entire course of training.

### NO ECONOMY IN HEALTH.

No economy in public health services is being tolerated by the Local Government Board, which is now insisting upon the carrying out of the regulations regarding measles. Islington Council has hesitated, feeling that work among children is better left to social and charitable concerns than to municipal administrations, but Whitehall has pointed out the imperative necessity of local authorities taking all practical steps to safeguard the health of young children. Moreover, failure to carry out the new regulations will involve the infliction of heavy penalties to the extent of £100, and a further penalty of £50 a day so long as the *laissez-faire* policy lasts. In these circumstances, Islington now proposes to appoint two health visitors, who will have to deal with about 2,500 cases of measles a year. The insistence on schemes of conserving young life will necessitate the employment of many health visitors throughout the country. The usual salary offered is £100 a year.

### A POINT FOR HOSPITAL SURGEONS AND MANAGERS.

Owing to the enormous quantities of iodine used as an antiseptic for wounds, both that substance and the salts from which it can be obtained are scarcer and more costly than they used to be. A correspondent points out to us that in many hospitals biniodide of mercury is largely used as an antiseptic for purposes which would be equally well served by corrosive sublimate (perchloride of mercury). This is a very timely hint for surgeons and secretaries of all institutions; the antiseptic value and other properties of these two substances are practically identical, and any individual preference for one over the other is to all intents and purposes a fad. In war time fads of this kind cease to be amiable; and we agree with the suggestion that the much cheaper perchloride should certainly be used in place of biniodide until the war is over. If this policy is carried out, not only will the institutions save money, but an iodine shortage in the treatment of wounds at the Front will be a contingency of diminished likelihood.

### THIS WEEK'S DRUG MARKET.

Apart from products which come within the category of drugs proper, two commodities which, though not generally classified as drugs, are commonly used in the manufacture of medicine are attracting special attention. These are spirits of wine and sugar. The fact, officially announced on February 15, that the large patent still distilleries are to be taken over by the Ministry of Munitions until the end of the war indicates that the shortage in the supply of rectified spirit which has been noticeable for some time will become accentuated, and it may be necessary to curtail very considerably the quantity of spirit used for medicinal purposes. Moreover, a further advance in the cost of spirit is most likely to take place. As regards sugar, the decision of the Government to restrict the imports and the advice given to consumers by the Royal Commission on the Sugar Supply to observe the strictest economy in the use of this product are matters which concern the drug departments of hospitals, as well as the stores departments generally. In the manufacture of medicinal syrups, compressed tablets, coated pills, medicated lozenges, and other preparations commonly prescribed in hospitals, it is difficult to see how the sugar bill can be cut down unless some alteration is made in the standard formulæ. But no doubt ways and means of effecting economy in the use of sugar without prejudice to the patients will occur to ingenious doctors and dispensers. In the drug market proper business has been less brisk during the week, but, on the whole, prices are well maintained, although there are few exceptions. Citric acid has again advanced in price as a result of a continuance of the large demand for export. Potassium permanganate is still dearer, and the scarcity is becoming very acute. The value of opium is well maintained, and any change in price would most probably be in an upward direction. Makers of iron and quinine citrate have raised their quotations. The price of ipecacuanha is not quite so firmly maintained, and santonin is slightly lower in value. Quinine is in less active demand, and the advance in price has ceased for the time being. Hexamine is again rather cheaper. At the recent public sale of drugs in Mincing Lane higher prices were paid for Cape aloes and honey, but cardamoms sold at slightly lower rates.

